# The trainees' perspective on developing an end-of-grant knowledge translation plan

**DOI:** 10.1186/1748-5908-5-78

**Published:** 2010-10-14

**Authors:** Brenda MY Leung, Cristina Catallo, Natalie D Riediger, Naomi E Cahill, Monika Kastner

**Affiliations:** 1 Department of Community Health Sciences, Faculty of Medicine, University of Calgary, Calgary, Alberta, Canada; 2 Daphne Cockwell School of Nursing, Ryerson University, Toronto, Canada, and European Observatory on Health Systems and Policies and the Centre for Health Economics and Policy Analysis, Department of Clinical Epidemiology and Biostatistics, McMaster University, Hamilton, Ontario, Canada; 3 Dept Community Health Sciences, University of Manitoba, Winnipeg, Manitoba, Canada; 4 Department of Community Health and Epidemiology, Queens University, Kingston, Ontario, Canada; 5 Department of Health Policy, Management, and Evaluation, University of Toronto, Toronto, Ontario, Canada

## Abstract

**Background:**

Knowledge translation (KT) is a rapidly growing field that is becoming an integral part of research protocols.

**Methods:**

This meeting report describes one group's experience at the 2009 KT Canada Summer Institute in developing an end-of-grant KT plan for a randomized control trial proposal.

**Results:**

Included is a discussion of the process, challenges, and recommendations from the trainee's perspective in developing an end-of-grant KT plan.

**Conclusion:**

New researchers should consider developing an end-of-grant KT plan with strategies that move beyond passive dissemination to incorporate innovative means of collaboration with the end user to craft the message, package the information, and share the research findings with end users.

## Introduction

Knowledge translation (KT) is a rapidly growing field that is becoming an integral part of research protocols. KT, as defined by the Canadian Institutes for Health Research (CIHR) is a complex, 'dynamic, and iterative process' comprised of synthesis, dissemination, exchange, and application activities in order to enhance the delivery and distribution of effective health care services [1]. Two models for KT are described by CIHR -- integrated and end-of-grant [2]. In an integrated KT model, researchers actively collaborate with potential end users through all stages of the research process from question generation, methods development, data collection and analysis, and/or dissemination of results [3]. End-of-grant KT focuses largely on dissemination activities at the end of a research project where messages are tailored for specific audiences and with various intensities from diffusion to dissemination to application [3,4] via traditional routes such as academic conferences and peer-reviewed journals to more innovative strategies to promote uptake of new knowledge such as through engaging the media [5]. CIHR has created a resource for researchers and trainees to facilitate the planning of effective end-of-grant KT activities. This guide includes the declaration of goals for dissemination, identification of a target audience, KT strategies, expertise and resources needed [4,6].

To enhance KT capacity, a training program in the form of a summer institute has been funded by CIHR. The second KT Canada Summer Institute (SI) was held in Toronto, ON, August 2009. The overall structure of the KTSI has been published elsewhere [7]. The focus of the 2009 KTSI was to explore the knowledge-to-action framework and expose trainees to opportunities and challenges in this field (Appendix 1). During the KTSI, trainees were assigned to small groups to work on various case studies from developing an end-of-grant KT plan to evaluating KT interventions used in research. Trainees worked collaboratively in their groups using a problem-based format supported by two or three KTSI faculty as facilitators. Our group was assigned to develop an end-of-grant KT plan under the guidance of our faculty facilitators (Drs. David Johnson, Sharon Straus, Sumit Majumdar) who were clinicians and academic researchers with experience in end-of-grant KT. To aid in completion of the task, we were provided with a document with 'tips for working successfully in a group' and some background reading associated with the task, namely: *Chapter 5 on Knowledge Dissemination and Exchange of Knowledge in Knowledge Translation in Health Care; CIHR End of Grant KT review document and checklist; *and *Summary of the Grant Proposal*. At the conclusion of the KTSI, each group presented their KT case assignment to the trainees and panel of KT experts.

This meeting report describes our group's experiences of developing an end-of-grant KT plan to be submitted as part of a CIHR grant proposal. The objectives of this meeting report are to: describe the process of developing an end-of-grant KT plan for a research proposal; explore the questions and challenges of this task; and provide recommendations for future end-of-grant KT plans.

### Process for developing an end-of-grant KT plan

Our group's KT case assignment was to create an end-of-grant KT plan for a randomized, double-blind controlled trial (RCT) to assess whether adding oxybutin to usual care of antimicrobial therapy would decrease pain and discomfort associated with childhood cystitis (Appendix 2). Because this was a grant proposal, an end-of-grant KT plan had to be created before study results were available.

The process of developing an end-of-grant plan involved first identifying our goal (*i.e.*,, to change practice versus increase awareness). Second, identifying the likely end users of the research results, and finally explicating the potential key messages for dissemination, and the principal target audience(s) and credible messenger(s) for each of these messages. This process of identification of our goals, audience, and message helped to inform the nature and intensity of the KT strategies to be selected from passive to active, such as: diffusion (*e.g.*, passive strategies such as peer reviewed publications and newsletters; dissemination (*e.g.*, tailor the message and medium to a particular audience; and application (*e.g.*, decision makers).

In order to guide decision making, our group created a template (Appendix 3) for developing an end-of-grant KT plan. This table permitted us to map out our goals, target audience, and KT strategies until we came to consensus through discussion. When developing our end-of-grant KT plan, a number of questions were generated that guided our discussion to arrive at consensus for the KT plan. See Appendix 4 for the guiding questions.

### Challenges to create an end-of-grant KT plan

The key challenges that arose for our group included the preliminary nature of the knowledge to be translated, resource limitations, and time allocated to operationalize the KT activities.

One major challenge was to identify a 'sufficient level' of evidence needed to change practice or influence decision making. Since our RCT was considered a preliminary study with a small sample size, the findings would require replication with a larger, more diverse sample before declaring confidence in its results. Thus, if we tried to engage a target audience of clinicians to change practice or clinician attitudes, findings from a single RCT would not be appropriate. Instead, dissemination strategies focused on crafting messages to fit within what else was known about the intervention and its effectiveness to build evidence for further research would be more appropriate.

An additional challenge faced by our group was the small proportion of the overall study budget directed to KT activities. This limitation meant that the scope of our strategy had to focus on passive modes of KT, such as traditional dissemination of results in journal publications and presentations at conferences, rather than more innovative (and expensive) strategies.

Finally, KT activities that require clinician participation or are time intensive may impose barriers to the dissemination of new research findings. Because our target audience included clinicians, we considered KT strategies that would not impose additional time restrictions, such as newsletters, downloads for personal electronic devices, and through network listserv newsflashes. However, future research activities could focus on active forms of dissemination (*e.g.*, workshops, web-based tutorials).

### Recommendations for end-of-grant KT plans

Our group identified a number of recommendations to assist KT trainees with the development of an end-of-grant KT plan. Based on this experience, our key recommendation is that an end-of-grant KT plan is not an 'add-on' for a grant proposal. Given time limitations of the SI setting and our lack of expertise in the substantive area of childhood cystitis, we found it difficult to develop a KT plan with clinical relevance (*i.e.*, the specific message to be translated), and to define the appropriate scope of the plan (*i.e.*, to identify the appropriate audience and strategies). Consequently, to enhance the learning experience we would strongly recommend that trainees be given the scenario and information prior to the SI meeting, and research into how best to develop a realistic and feasible end-of-grant KT plan. Perhaps provide the opportunity for trainees to consult clinical researchers, KT experts, and end users so that there is adequate time to engage fully in the KT development process.

Another key is to assign a specific portion of the grant budget to KT activities, as end-of-grant KT plans are often limited by the amount of grant funding awarded for the overall project. This would allow for more innovative approaches to end-of-grant KT plans to engage representatives of the target audience to help craft key messages so that the research findings are accepted as both credible and relevant for the end user. This would involve developing strategies as part of the KT plan related to how the information is packaged and shared with end users. A key lesson for our group was that the methods in which information was packaged and shared with end users, such as clinicians, may have a role in facilitating uptake of research evidence.

## Conclusion

Feedback from the SI attendees and KT experts was that our plan (see Appendix 1) was too complex and extensive for a small component of the grant proposal. However, the KT experts emphasized the importance of a KT component to facilitate a successful grant application. The dichotomous nature of this feedback underscores the need for more clear and comprehensive goals and guidelines for researchers from granting agencies, and to consider the limitations of various approaches of including a KT plan in a research protocol. If granting agencies mandate the inclusion of KT plans in the proposal, then they might consider providing resources that will enable the full realization of the KT piece. These might include: a process for connecting new researchers with KT expertise; providing sufficient funds for a well conceived and practical KT plan; and allowing for a more realistic KT plan (*i.e.*, varying size and scope) relative to the proposal, rather than as a formula preconceived by the agency. We believe that these suggestions might enable a broader uptake of KT in research, and to positively impact sharing and translating of knowledge.

## Competing interests

The authors declare that they have no competing interests.

## Authors' contributions

BL developed the format for the paper, collated the information, and coordinated the content from various authors, as well as edited and formatted the final draft. CC assisted with early versions of the draft and provided revisions to the final draft. NDR and NEC contributed a portion of the content and minor editing revisions.

All authors have read and approved the final manuscript.

## Appendix 1. Overview of CIHR Summer Institute in Knowledge Translation 2009

### Purpose

To provide participants with the opportunity:

1. to increase their understanding of knowledge translation research as well as opportunities and challenges in this field.

2. to network with colleagues and national and international mentors.

### Theme

Exploring the Knowledge to Action Framework

### Attendees

Thirty graduate students, postdoctoral, and clinical fellows enrolled at a Canadian Institution studying issues relevant to knowledge translation.

### Objectives

1. Explore the challenges of planning and completing KT research.

2. Gain better understanding of the research gaps in the KT field.

3. Explore the knowledge to action framework, its role in advancing the science of KT and some research gaps within this framework.

4. Investigate the contribution of different disciplinary and methodological approaches for KT research within the knowledge to action framework.

5. Network with other young researchers interested in KT research and KT as well as with mentors experiences in the science and practice of KT

6. Experience a supportive training environment that is respectful of the perspectives, tools and approaches of all disciplines.

### Agenda

#### Day 1

1. Welcome: Introduction and overview of the Summer Institute Sharon Straus, University of Toronto

2. Plenary: Introduction to knowledge translation and overview of research gaps Jacqueline Tetroe, Canadian Institues of Health Research

3. Small group session^a^

4. Plenary: Knowledge tools: Patient decision aids Dawn Stacey, University of Ottawa

5. Small group session

6. Meet the Faculty

#### Day 2

1. Plenary: KT tools: Clinical practice guidelines and their adaptation and implementation Melissa Brouwers, Cancer Care Ontario

2. Plenary: Barriers and Facilitators France Légaré, Universite Laval

3. Small group session

4. Plenary: Selecting KT interventions Sumit Majumdar, University of Alberta

5. Small group session

6. Meet the Faculty

#### Day 3

1. Plenary: Evaluating KT interventions: qualitative and quantitative, Martin Eccles, University of Newcastle

2. Presentations from small groups

3. Conclusion and evaluation Sharon Straus, University of Toronto

^a ^There were a total of five small groups with six trainees in each group. Each group was assigned a different case study. The topics of the other four cases were: Developing a KT intervention for multiple stakeholders; Testing a KT theory; Developing a consensus statement on barriers; and Evaluation of a KT intervention

## Appendix 2. Description of the study

### Description of study

#### Title

Efficacy of Oxybutynin in Paediatric Cystitis

### Purpose

To determine if the addition of the bladder antispasmotic oxybutynin to standard antimicrobial therapy in the treatment of childhood cystitis will decrease the associated pain and discomfort.

### Design

Randomized, double blind, placebo-controlled clinical trial

### Rationale

Urinary tract infections (UTIs) are common among the paediatric population. Research indicates painful symptoms of UTIs among adults, which are managed with medication. There is little research regarding the incidence of UTI symptoms among children or approaches to management. Oxybutynin is used for management of other non-infectious bladder conditions among children and has an established safety profile.

### Population

Toilet trained children aged 4 to 16 years old presenting at the Emergency room with a diagnosis of cystitis.

### Outcomes

1. Self-reported pain

2. Survey of symptoms

## Appendix 3. End-of-grant KT plan

### Goals

1. Increase knowledge/awareness

a. Nature and duration of symptoms specific to children

b. Effectiveness of intervention in reducing pain/discomfort in children with cystitis

2. Inform future research

### Audience

1. Healthcare Practitioners including emergency physicians, pediatricians, pharmacists, nurses

2. Researchers

3. Other, *e.g.*, media, parents, study participants

### Strategies for diffusion

1. Publish results in peer review journal (*e.g.*, medical association journals, paediatric , urology, emergency med)

2. Conferences - continuing medical education (CME) topics

3. Study specific website

4. Non-peer review publications

a. Parenting magazines

b. Websites *e.g.*, medical associations/societies,

c. Downloadable e-info (*e.g.*, PDA/DVD, instant messaging)

d. Association/society publications by specializations

### Strategies for dissemination

1. Involve end-users in developing the message for use in a CME module:

a. Convene meeting where health care practitioners (HCP) are involved in crafting key messages related to the study results for use in a CME module

b. Form focus group

i. Look at attitudes - measure HCP attitudes toward the intervention implementation; identify barriers/facilitators to implement intervention

ii. Involve users in determining messages from study/how to best reach users

c. Survey HCP motivation to use intervention/results; assess attitudes toward intervention

2. Involve champions - after CME module is created, identify motivated clinicians who can serve as 'trainors' for the CME module and address potential barriers/facilitators to implementing study results

3. Education - present results using a variety of educational opportunities

a. Structured abstracts→ accessible, ACP Journal Club, *et al*.

b. Grand rounds

c. CME credit

d. Newsletters - web-based

4. Decision aids → provide consistency for screening

## Appendix 4. Guiding questions for group discussion

### 1. Who is the target audience and how to best engage them?

Given limited KT resources, modes of dissemination require the researcher to consider where and how the information will be disseminated. While the study focused on children with cystitis, we decided that clinicians (primarily emergency physician and pediatricians) would be our primary audience because they would most likely be influenced by a potential practice change should this trial indicate a preferred treatment strategy.

### 2. What are the most impactful KT strategies that can be used?

That is, how can we best focus limited resources for maximum impact given a particular target audience? Because a small proportion of an operating grants budget is allotted to the KT plan, we decided to focus on strategies aimed at diffusion and dissemination. For diffusion, we aimed to publish study results using traditional methods such as academic journals and conferences. However, in order to facilitate greater engagement with study results, we wanted to consider active dissemination, such as involving the target audience in crafting the message for use in an education strategy, such as a CME activity.

### 3. What is a realistic plan that is feasible, economical, and effective?

Likely, multiple strategies would be identified and required depending on the audiences. When we identified a potential KT strategy for use in the end-of-grant plan, we created a matrix that outlined the facilitators and barriers for each strategy. This permitted us to prioritize the various activities according to their potential impact, feasibility, and cost, thus enabling the group to achieve consensus regarding the selection of the most appropriate KT strategies.
